# An Improvement of Mechanical Properties of Two Kinds of Silicone Resins Containing Ladder Segments by Chemical Modification with Trimethylborate

**DOI:** 10.3390/ma16083072

**Published:** 2023-04-13

**Authors:** Alexei V. Kalinin, Vjacheslav V. Zuev

**Affiliations:** 1 S.V. Lebedev State Institute of Synthetic Rubber, 1, Gapsalskaya St. 1, 198035 Saint Petersburg, Russia; 2 Chemical Engineering Centrum, ITMO University, Kronverkskiy Pr. 49, 197101 Saint Petersburg, Russia; 3 Institute of Macromolecular Compounds of the Russian Academy of Sciences, Bolshoi Pr. 31, 199004 Saint Petersburg, Russia

**Keywords:** silicone, coating, nanostructure, postsynthesis modification

## Abstract

We suggest a new method for postsynthesis modification of silicones containing silanol groups. It was found that trimethylborate is an effective catalyst for dehydrative condensation of silanol groups with the formation of ladder-like blocks. The utility of this approach was demonstrated on postsynthesis modification of poly-(*block* poly(dimethylsiloxane)-*block* ladder-like poly(phenylsiloxane)) and poly-(*block* poly((3,3′,3″-trifluoropropyl-methyl)siloxane)-*block* ladder-like poly(phenylsiloxane) with a combination of linear and ladder-like blocks having silanol groups. The postsynthesis modification leads to a 75% increase in tensile strength and 116% elongation on break in comparison with the starting polymer.

## 1. Introduction

The silicone rubbers are widely used as inert materials and coatings thanks to their excellent properties [1]. However, any advantages of these materials, such as low surface energy, have drawbacks, such as low adhesion and mechanical performance [2]. One way to improve these properties is through the chemical modification of these polymers and the postsynthesis increase in their molecular weight [3]. The synthesis of highly controlled silicone materials that meet economic and sustainability requirements is extremely coveted [4]. The most common approach to postsynthesis modification of silicone polymers is the hydrosilylation reaction, using various catalysts based on metal-organic systems [5]. Most of these catalysts are very expensive [6].

The popular method for improving the mechanical properties of polymers is the preparation of composites using fillers of various dimensions, including micro- and nanosized fibers and powders [7]. As an alternative, we suggest the synthesis of silicone-based multiblock copolymers with linear and ladder blocks, in which the ladder blocks act as nanofibers, leading to self-reinforcement of the material [8]. Unfortunately, the chosen synthetic pathway leads to the formation of many defects in ladder blocks. As a result, the adhesion and mechanical properties of the obtained block copolymers are unsatisfactory. The defects of ladder blocks are open cycles with free silanol units. Hence, the postsynthesis modification of these multiblock copolymers should include dehydration with cyclization to obtain the defect-free ladder blocks.

In 1978, Ganem et al. reported that carboxylic acids react with amines in mild conditions in the presence of 1,3,2-dioxaboranes (**I**) generated in situ [9]. Later, the catalytic activity in the dehydrative amide condensation was found for 1,3,2-dioxaboralanes (**II**). These catalysts are synthesized in situ from polyols and boronic acid or alkylboranes [10,11]. The boronic acid and alkylboranes are available at low prices, especially in comparison with the hydrosilylation catalysts [12]. The multiblock copolymers containing linear polydimethylsiloxane (**III**) or polymethyltrifluoropropylsiloxane (**IV**) and ladder-like polyphenylsiloxane blocks synthesized previously by us contain silanol groups in ladder-like blocks, which can produce 1,3,2-dioxaboralones (**II**) or 1,3,2-dioxaboranes (**I**) in reaction with boronic acid or alkylboranes. Hence, the catalyst for cyclic dehydration of silanol groups can be prepared in situ.

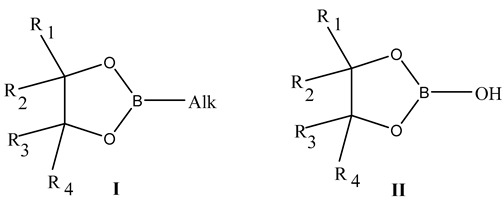



The aim of the present study is to develop a method of postsynthesis modification of silicone-containing linear-ladder multiblock copolymers for improving their adhesion and mechanical properties.

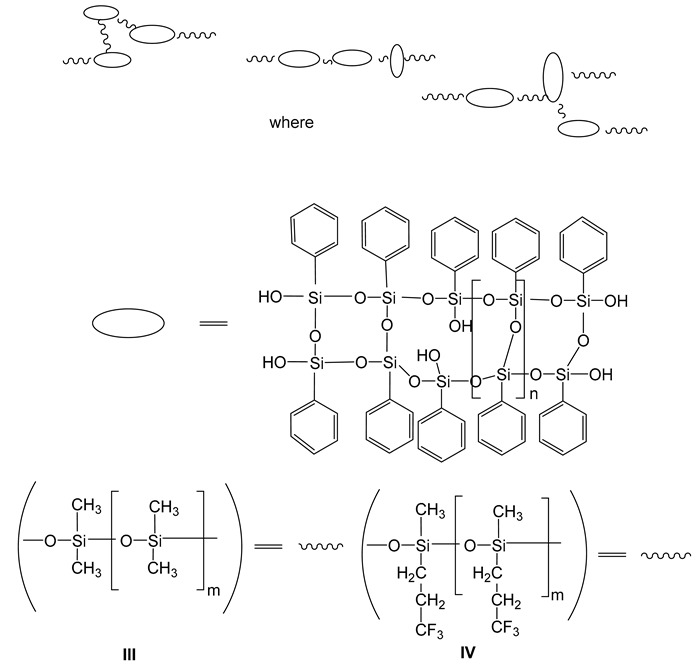



## 2. Experimental Part

Polymers **III** and **IV** were prepared as described previously [8]. All solvents and reagents were purchased from Reachim (Saint Petersburg, Russia) and distilled or recrystallized before use.

FTIR spectra were recorded on a Vertex 50 FTIR (Bruker, Billerica, MA, USA) with ATR sampling accessor.

^11^B and ^29^Si solutions NMR spectra were recorded on an AVANCE 400 (Bruker) spectrometer. Spectra were recorded in CDCl_3_ solution. Data were recorded as follows: chemical shift in ppm from internal tetramethylsilane (for ^29^Si δ = 0) or boronic acid (for ^11^B δ = 20.0) on the scale δ.

Gel permeation chromatography, mechanical and adhesion testing were conducted as described in Supplementary Materials.

### 2.1. Postmodification Procedure

A round-bottom flask equipped with a teflon-coated magnetic stirring bar and a reflux condenser was charged with 100 g of copolymer **III** in chlorobenzene (250 mL). After polymer dissolving, 1.24 g (~2 × 10^−2^ mol) of trimethylborate was added and the solution was heated at reflux for 16 h. After that, approximately 120 mL of chlorobenzene was distilled off, and the remaining solution was used for film casting. The films were dried in vacuo. 

### 2.2. Method of Determination of Si-OH Groups in Silicone Resins

**Figure 1:**
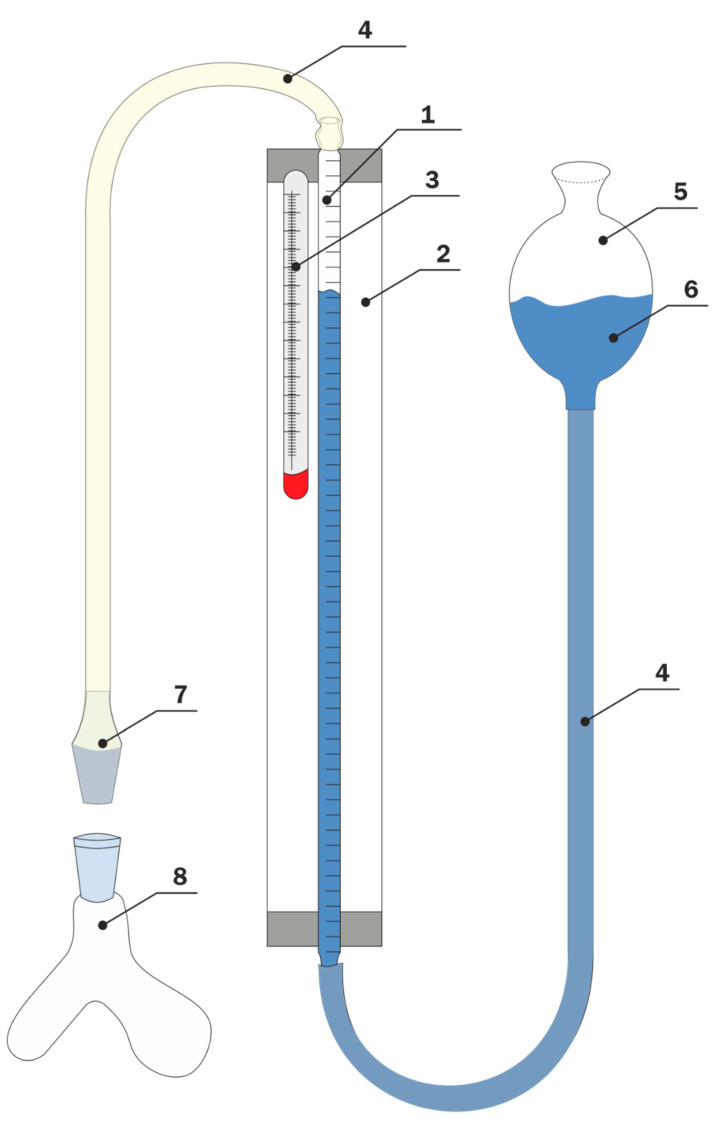
Schematic representation of the laboratory instrument for the determination of Si-OH group content. 1—gas burrete, 2—glass thermostat, 3—thermometer, 4—silicone hosepipe, 5—filler, 6—barrier fluid, 7—adapter, 8—special two-necked flask.

For the determination of Si-OH groups contained in the silicone under study, we use the reaction of methylmagnesium iodine (Grignard reagent) with hydroxyl-containing substrate with evaluation of methane (Zerevitinov Test [13]). This gas can be determined quantitatively by measuring its volume using the instrument presented in Figure 1. As barrier fluid (6 in Figure 1), the oligomers of trifluorochlorineethylene (polymerization grades 5–10) were used. The Grignard reagent was prepared in dibuthyl ether. The hitch of silicone was dissolved in toluene for copolymer III or chlorobenzene for copolymer IV and put in a glass container or flask. On the other arm of this flask, 5–10 mL of solution of Grignard reagent with a quantitatively determined concentration was added. After the blowing of all apparatus with inert gas (Ar), the solution was mixed in a special two-necked flask (8) and the volume of methane was measured. The Zerewitinoff determination usually takes 5–30 min and provides results with accuracy and reproducibility of ±3–5%.

## 3. Results and Discussion

The condensation of trichlorophenylsilane in water with the formation of ladder polymers leads to the formation of products with many defects, such as open units with silanol groups [1]. As was shown in our previous paper using solid-state ^29^Si NMR spectroscopy, the block-copolymer **IV** with trifluoropropyl substituents in linear blocks contains more defects than its analogs **III** with polydimethylsiloxane linear blocks [8]. However, the solid state ^29^Si NMR spectroscopy did not allow the quantitative determination of the amount of silanol groups because of the long relaxation time of ^29^Si nucleus in the NMR experiment [14]. Therefore, for the quantitation of silanol group amount, we used the average OH number, which was determined by the Zerewitinoff method. For block-copolymer **IV,** we found that the average OH number is approximately 6–11 mg KOH/g. For block-copolymer **III**, this number is 0.7–1.1 mg KOH/g. Such differences in the average OH number values for block copolymers obtained by similar methods can be explained by differences in compatibilities between ladder-like poly(phenylsiloxane) blocks and poly(dimethylsiloxane) blocks or poly(poly((3,3′,3″-trifluoropropyl-methyl)siloxane) blocks. The latter are less compatible with ladder blocks, which leads to a less dense conformation of block-copolymer **IV** in solution at synthesis and, as a result, the formation of more defects in ladder blocks because the reaction of condensation is reversible [8].

The presence of defects in ladder blocks leads to a decrease in their ability to reinforce the polymer matrix, as happens with nanosized fibers or rods. Hence, the removal of these defects should improve the mechanical and adhesion properties of the polymers in the study. As a method for such transformation, we selected the reaction that is similar to the condensation of organic acids with amines catalyzed by cyclic 1,3,2-dioxaboranes discovered by Ganem et al. in 1978 [9].

**Figure 2:**
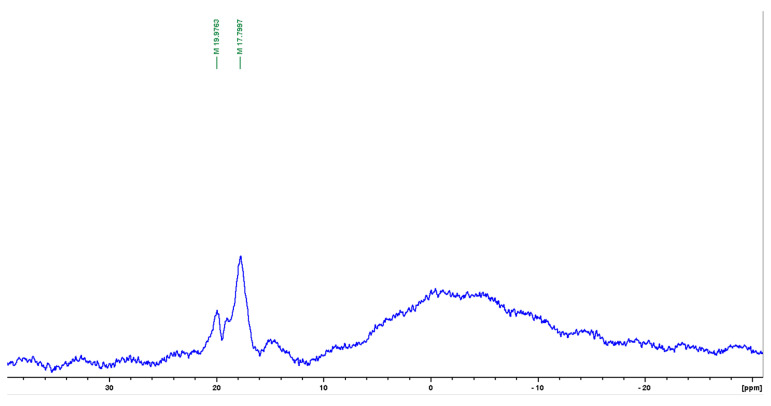
^11^B NMR spectrum of copolymer **III** in solution of chloroform-d.

The presence of silanol groups in the ladder-like blocks of copolymers **III**–**IV**, which can react with boranes to form cyclic1,3,2-dioxaborane structures, encouraged us to try this pathway. The advantages of this approach are the following: the use of the cheapest reagent (trimethylborate) in a minimal amount with the in situ formation of 1,3,2-dioxaborane as a catalyst of silanol condensation; the absence of the need for purification from this catalyst and the mild condition of the reaction. The solution of copolymers **III** or **IV** in chlorobenzene with a small amount of trimethylborate was brought to reflux for 3 h, and then the solvent was distilled out. The resulting polymers form transparent films. The formation of 1,3,2-dioxaborane **V** was confirmed by ^11^B NMR spectroscopy (Figure 2). The signal at 17.8 ppm in the ^11^B NMR spectrum of copolymer **III** indicates the formation of 1,3,2-dioxaborane **V** [15]. Hence, we observe the formation of a potential catalyst.

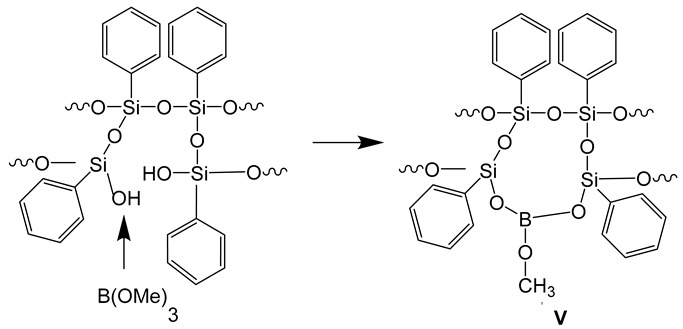



We investigated the properties of copolymers **III**–**IV** after heating their solutions in the presence of trimethylborate. The molecular weights of these copolymers before modification determined by liquid chromatography were for **III** M_w_ = 9.0 × 10^4^, M_w_/M_n_ = 1.97 and for **IV** M_w_ = 4.2 × 10^4^, M_w_/M_n_ = 2.32. After modification, the apparent molecular weight of copolymer **III** increases up to M_w_ = 1.0 × 10^5^ and for copolymer **IV** up to M_w_ = 5.1 × 10^4^. This increase can be seen because we used a polystyrene standard for calibration and an increase in polymer chain rigidity should lead to overestimated molecular weights determined using liquid chromatography [16].

To support the catalytic impact of the formed 1,3,2-dioxaborane **V**, we conducted the experiment by heating the copolymer **IV** without adding trimethylborate. As one can see from the liquid chromatography data (see Supplementary Materials, Figures S5 and S6), nothing happens with copolymer IV after heating at 132 °C in chlorobenzene for 6 h. Other support for the catalytic activity of 1,3,2-dioxaborane **V** is the absence of gel fraction formation at postmodification by adding trimethylborate. In the case of B(OCH_3_)_3_, only reactions with the silanol groups of the silicone resin and some boron atoms incorporated in the structure of modified silicone resins should also form the interchain O-B-O bridge. Hence, the formation of gel-fractions. Therefore, these results, in combination with literature data, provide a reason for the manifestation of catalytic activity of formed 1,3,2-dioxaborane V in the condensation of silanol groups.

**Figure 3:**
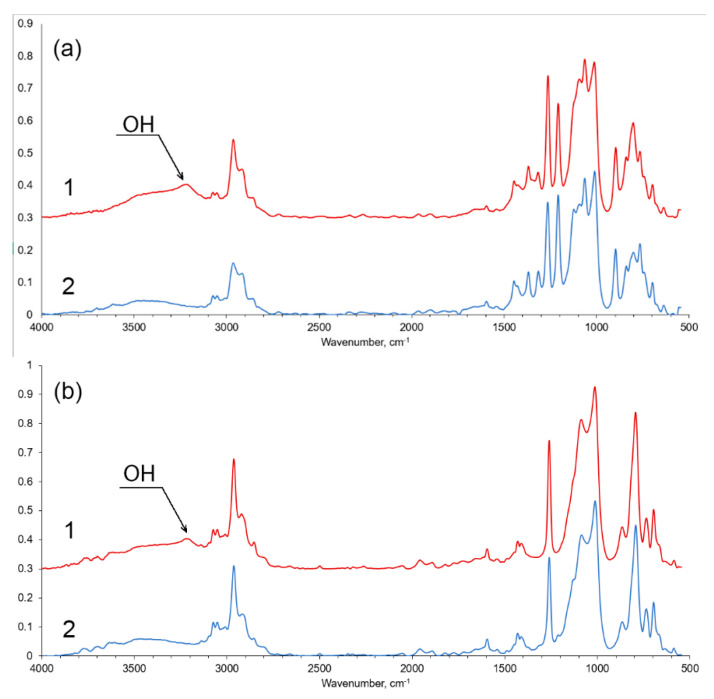
FTIR spectra of neat copolymer **IV** (1) and after modification (2) (**a**); of neat copolymer **III** (1) and after modification (2) (**b**).

We estimated the average OH number for modified copolymers **III**–**IV**. For modified copolymer **III,** this value is 0.3–0.4 mg KOH/g, and for copolymer **IV,** it is 2.5–3.0 mg KOH/g. Hence, the containment of silanol groups decreased by factors of two and four. This observation was supported by the results of IR spectroscopy (Figure 3). After treatment, the signals of silanol groups disappeared from the FTIR spectra of copolymers **III**–**IV** (Figure 3). The signals of silanol groups are absent in the solution ^29^Si NMR spectra of these copolymers (see Supplementary Materials, Figure S2). Hence, as a result of this modification, the ladder blocks lost most of their defects and were converted to rigid rods.

These results allow us to study the mechanical properties of modified copolymers **III**–**IV** (see Supplementary Materials). The tensile strength of modified copolymer **III** increases to 1.61 ± 0.07 MPa from 0.92 ± 0.22 MPa for the starting polymer (an increase of 75%). The elongation on break increases by 116% from 76.7 ± 22.2 to 165.8 ± 17.3. The mechanical properties of copolymer **IV** are poor, and the tensile strength of both the starting and modified polymers is less than 50 kPa. It is impossible to perform a test using dumbbell-shaped specimens on a breaking machine. The motivation for such behavior leads to interactions between the chains of block copolymers under study. The fluorine-containing chains of copolymer **IV** did not interpenetrate in hard domains formed by ladder-like units [17]. It leads to fluidity in the material. Polydimethylsiloxane chains interpenetrate in the hard domains and form ladder-like segments [17]. As a result, copolymer **III** shows high mechanical performance.

The improvement in the mechanical performance of copolymer **III** leads to an increase in adhesion strength. The adhesion to cooper increases from 0.71 MPa to 1.60 MPa, to aluminum from 0.96 MPa to 1.78 MPa, to steel from 1.70 MPa to 2.74 MPa, with deviations from sample to sample within 0.15 MPa. The adhesion of copolymer **IV** to all materials is less than 0.4 Mpa because of its poor mechanical performance.

For the measurement of the water contact angles of copolymers **III** and **IV**, their solutions in methylene chloride were cast on supporting plates from steel, copper or aluminum, after which they were tempered at 70 °C. Since this temperature was much higher than the glass transition temperatures of III and **IV**, using solvent did not play any role in the surface formation of the resulting films. For copolymer **III**, the measured water contact angles of films for both starting and modified copolymers did not depend on substrate materials. The contact angle was 107.5° ± 1.1°.

For modified copolymer **IV**, the water contact angle increases from 110.5° to 111.0° on aluminum, from 111.3° to 112.4° on steel and from 115.7° to 117.0° on copper, with a mean deviation of 1°. Hence, the postmodification of silicone materials significantly changes their mechanical performance while having little effect on their surface properties.

The approach for chemical postmodification of polysiloxanes based on using a catalyst with Lewis acid properties [18] was suggested by us. The boranes as reagents offer a variety of different outcomes to the modification of the polysiloxanes with generations of complex architectures [19]. Such metal-free catalysts are environmentally friendly [20,21,22,23,24,25,26]. The condensation of silanol groups into ladder-like units has been shown to be an excellent way to create self-reinforced nanocomposites.

## 4. Conclusions

The production of ladder silicones via the condensation of chlorosilanes in water leads to polymers with many structural defects, such as the formation of open units in ladder chains with silanol groups. We have shown that trimethylborate is an economically and practically efficient catalyst for interchain dehydrative condensation of such silanol groups, resulting in defect-free ladder silicones. This approach was demonstrated for the postsynthesis modification of siloxane-based block copolymers **III** and **IV**. The suggested method allows for significant improvements in the mechanical performance of these polymers. The tensile strength of copolymer **III** was increased by 75% and the elongation on break by 116% in comparison with neat polymer after condensation synthesis. The presence of silanol groups is a common feature of siloxane polymers obtained by condensation of dichlorosilanes, and a suggested method can be used to improve their mechanical properties.

## Data Availability

The other data are not available.

## Supplementary Materials

The following supporting information can be downloaded at: https://www.mdpi.com/article/10.3390/ma16083072/s1, Figure S1. The films of modified copolymers **III** (1) and **IV** (2). Figure S2. 29 Si NMR spectrum of copolymer III after modification in chloroform- d solution. Figure S3. Mechanical testing of copolymer III before modification. Figure S4. Mechanical testing of copolymer III after modification. Figure S5. The chromatogram of copolymer **IV** before heating (starting copolymer). Figure S6. The chromatogram of copolymer IV after heating at 132 °C in chlorobenzene at 6 h.

## References

[1] Witucki G.L., Tator K.B. (2015). Polysiloxane Hybrid Coatings. Protective Organic Coatings.

[2] Hellio C., Yebra D. (2009). Advances in Marine Antifouling Coatings and Technologies.

[3] Zuev V.V., Smirnova G.S., Nikonorova N.A., Borisova T.I., Skorokhodov S.S. (1991). Chemical modification of polymer main chain—A new path to liquid-crystalline polyesters with long siloxane segments. Significance of molecular mobility in the formation of the liquid-crystalline state. Makromol. Chem..

[4] Muzafarov A.M. (2011). Silicon Polymers.

[5] Marciniec B. (2009). Hydrosilylation: A Comprehensive Review on Recent Advances.

[6] Troegel D., Stohrer J. (2011). Recent Advances and Actual Challenges in Late Transition Metal Catalyzed Hydrosilylation of Olefins from an Industrial Point of View. Coord. Chem. Rev..

[7] Zuev V.V. (2011). Polymer Nanocomposites Containing Fullerene C60 Nanofillers. Macromol. Symp..

[8] Ostanin S.A., Kalinin A.V., Bratsyhin Y.Y., Saprykina N.N., Zuev V.V. (2021). Linear/Ladder-like Polysiloxane Block Copolymers with Methyl-, Trifluoropropyl- and Phenyl-Siloxane Units for SurfaceModification. Polymers.

[9] Collum D.B., Chen S., Ganem B. (1978). A new synthesis of amides and macrocyclic lactams. J. Org. Chem..

[10] Maki T., Ishihara K., Yamamoto Y. (2006). 4,5,6,7-Tetrachlorobenzo[d][1,3,2]dioxaborol-2-ol as an Effective Catalyst for the Amide Condensation of Sterically Demanding Carboxylic Acids. Org. Lett..

[11] Charville H., Jackson D., George Hodges G., Whiting A. (2010). The thermal and boron-catalysed direct amide formation reactions: Mechanistically understudied yet important processes. Chem. Commun..

[12] Liu J., Lavigne J.J., Yall D.G. (2011). Boronic acids in material chemistry. Boronic Acids: Preparation and Applications in Organic Synthesis, Medicine and Materials, 1&2.

[13] Wang Z. (2010). Zerewitinoff Determination. Comprehensive Organic Name Reactions and Reagents.

[14] Uhlig F. (2017). ^29^Si NMR Spectroscopy. Organosilicon Compounds.

[15] Lewiński J., Kubicki D. (2017). NMR Spectroscopy, Heteronuclei, B, Al, Ga, In, Tl. Encyclopedia of Spectroscopy and Spectrometry.

[16] Tsvetkov V.N., Tsvetkov N.V., Zuev V.V., Didenko S.A. (1995). The effect of the length of flexible chain fragments on electrooptical properties of mesophase formed by chain molecules. Vysokomol. Soed. Ser. A B.

[17] Ostanin S.A., Mokeev M.V., Zuev V.V. (2022). Influence of Interpenetrating Chains on Rigid Domain Dimensions in Siloxane-Based Block-Copolymers. Polymers.

[18] Yamamoto H. (2000). Lewis Acids in Organic Synthesis.

[19] Gao H., Battley A., Leitao E.M. (2022). The ultimate Lewis acid catalyst: Using tris(pentafluorophenyl) borane to create bespoke siloxane architectures. Chem. Commun..

[20] Vogel P., Lam Y., Simon A., Houk K.N. (2016). Organocatalysis: Fundamentals and Comparisons to Metal and Enzyme. Catalysts.

[21] Mokeev M.V., Ostanin S.A., Zuev V.V. (2019). Prototropic behavior of cyclohexane substituted urethane and urea compounds. Observation of H-bond mediated ^4H^J_H1H3_ coupling constants across urea fragments. Tetrahedron.

[22] Dharmaratne N.U., Pothupitiya J.U., Bannin T.J., Kazakov O.I., Kiesewetter M.K. (2017). Triclocarban: Commercial Antibacterial and Highly Effective H-Bond Donating Catalyst for Ring-Opening Polymerization. ACS Macro Lett..

[23] Fastnacht K.V., Spink S.S., Dharmaratne N.U., Pothupitiya J.U., Datta P.P., Kiesewetter E.T., Kiesewetter M.K. (2016). Bis-and Tris-Urea H-Bond Donors for Ring-Opening Polymerization: Unprecedented Activity and Control from an Organocatalyst. ACS Macro Lett..

[24] Pothupitiya J.U., Hewawasam R.S., Kiesewetter M.K. (2018). Urea and Thiourea H-Bond Donating Catalysts for Ring-Opening Polymerization: Mechanistic Insights via (Non)linear Free Energy Relationships. Macromolecules.

[25] Lin B., Waymouth R.M. (2017). Urea Anions: Simple, Fast, and Selective Catalysts for Ring-Opening Polymerizations. J. Am. Chem. Soc..

[26] Auvil T.J., Schafer A.G., Mattson A.E. (2014). Design Strategies for Enhanced Hydrogen-Bond Donor Catalysts. Eur. J. Org. Chem..

