# Supplementary material for: An Improvement of Mechanical Properties of Two Kinds of Silicone Resins Containing Ladder Segments by Chemical Modification with Trimethylborate
Source: Materials (Basel). 2023 Apr 13;16(8):3072. doi: 10.3390/ma16083072 (PMC10144118; doi:10.3390/ma16083072)
Supplement: Supplementary file 1 [file materials-16-03072-s001.zip › materials-2319087-supplementary.pdf]

Description of testing methods

Gel permeation chromatography was conducted on a Breeze QS HPLC (Waters) equipped with three different columns connected in series (HR-1, HR-2 and HR-4). Two detectors were employed in this study, a UV (Ultraviolet) Waters 2487 operated at a wavelength of 254 nm and differential refractive index Waters 2414. Tetrahydrofuran (THF) was used as eluent at a flow rate of 0.35 mL/min at 35 °C. The system was calibrated using polystyrene standards with different Mw obtained from Sigma-Aldrich using the universal calibration method.

Mechanical properties of the copolymer **III-IV** such as tensile strength and elongation at break were measured according to ISO 527-2 using the Universal Testing Machine Instron 5966 with sample dimensions: 0.1 x 0.01 x 0.00035 m. At least 5 samples were used for each composition.

Adhesion of copolymers to metals was tested according to ISO 4624:2002 by the destructively testing of the bonded coupons using PosiTest AT-M Manual Adhesion Tester (DeFelsko, USA). Average of 5 measurements is reported.

Drop Shape Analyzer DSA100E KRUSS was employed to study the contact angle of a water droplet on the block-copolymers films by the sessile drop method. Average of 5 measurements taken on polymer samples coated on 5 separate metal substrates is reported as the water contact angle.

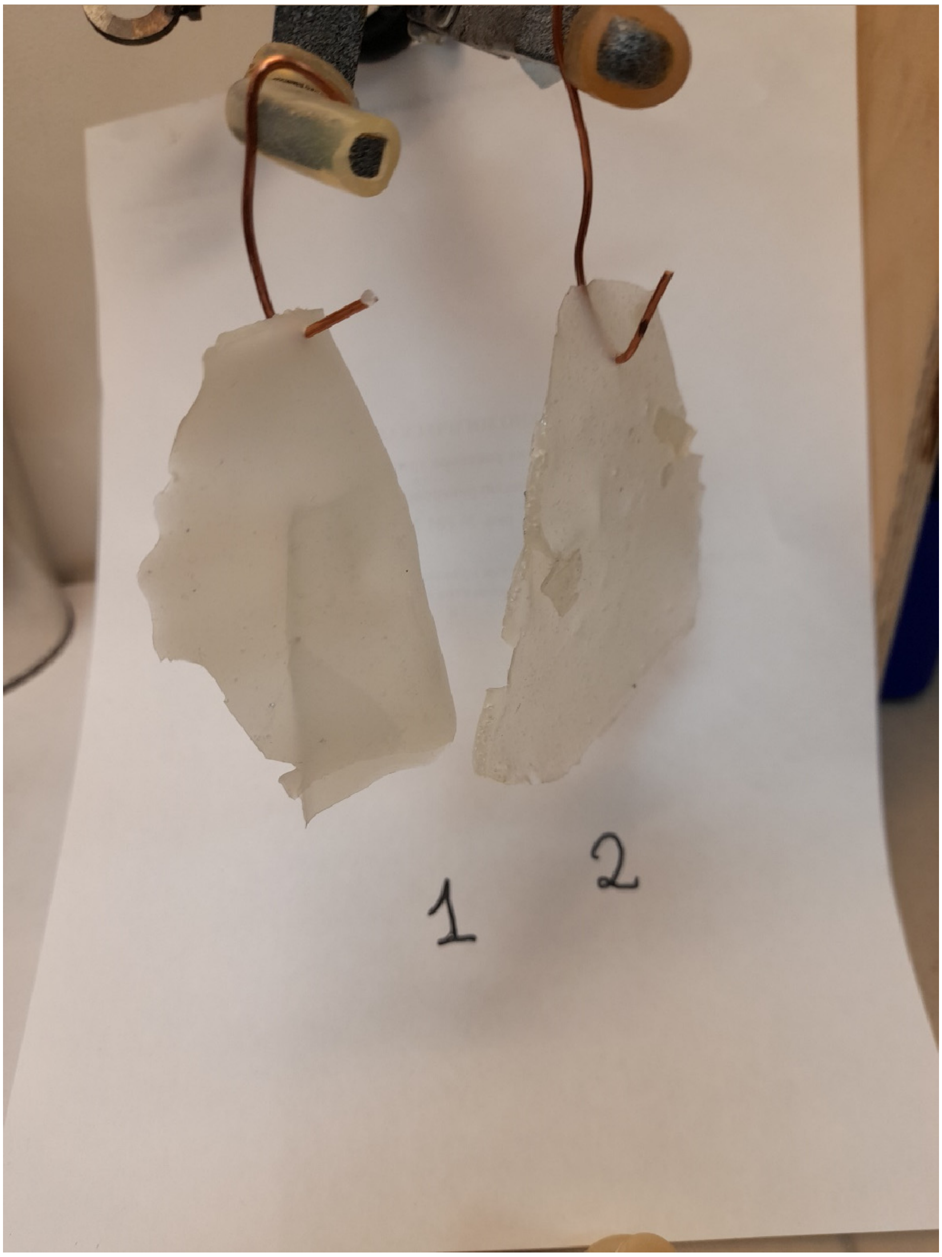

Figure S1. The films of modified copolymers **III** (1) and **IV** (2).

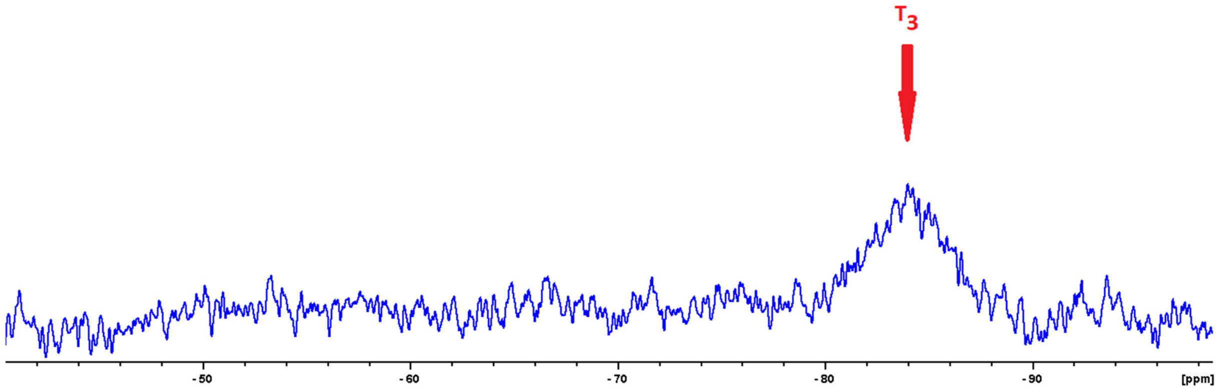

Figure S2. 29 Si NMR spectrum of copolymer III after modification in chloroform- d solution.

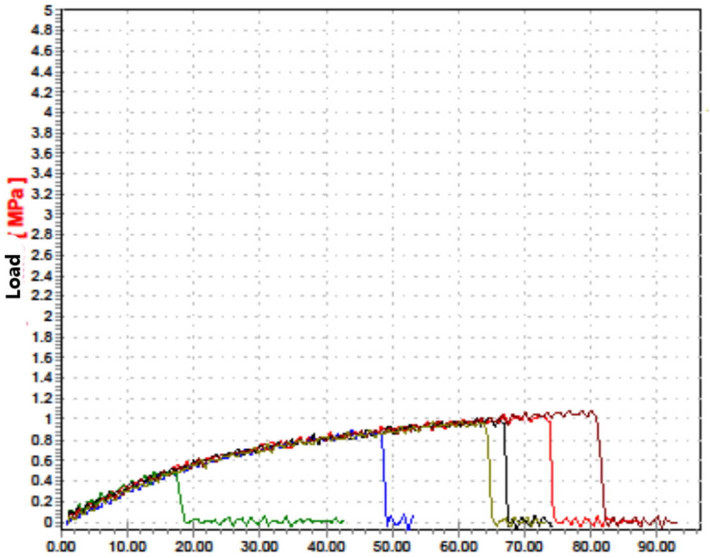

Figure S3. Mechanical testing of copolymer III before modification

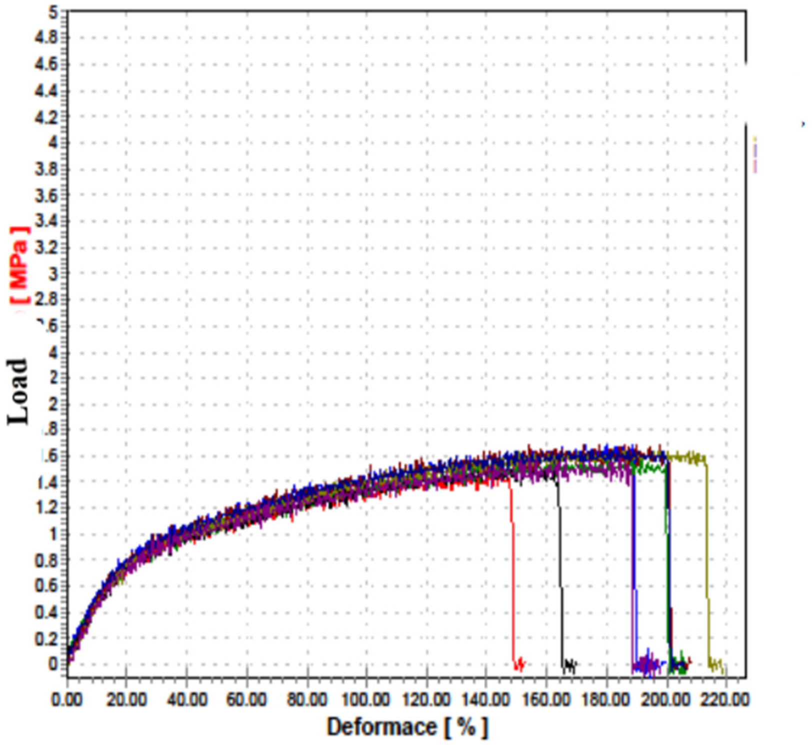

Figure S4. Mechanical testing of copolymer III after modification

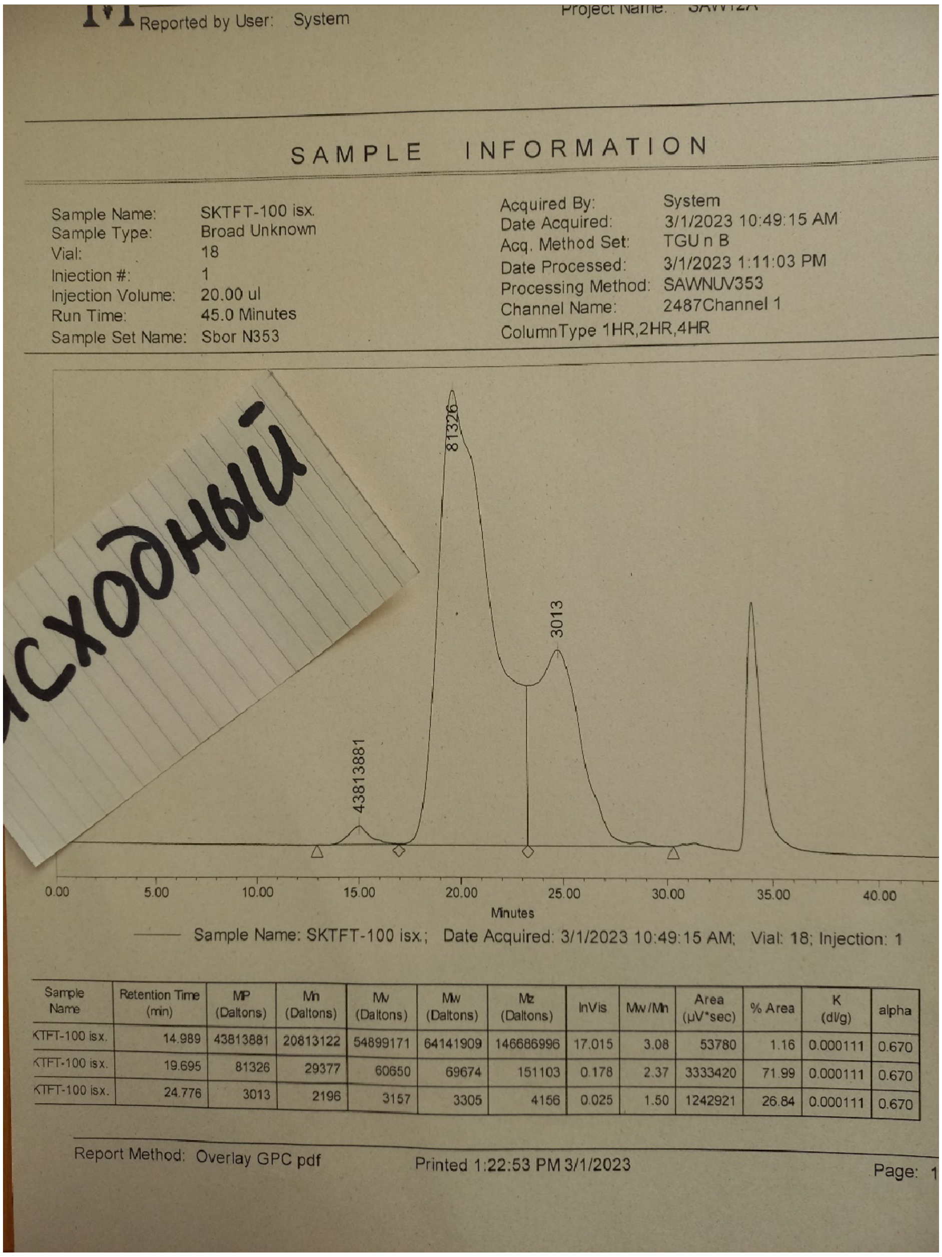
Reported by User: System

Project Name: [illegible]

SAMPLE INFORMATION

Sample Name: SKTFT-100 isx.
Sample Type: Broad Unknown
Vial: 18
Injection #: 1
Injection Volume: 20.00 ul
Run Time: 45.0 Minutes
Sample Set Name: Sbor N353

Acquired By: System
Date Acquired: 3/1/2023 10:49:15 AM
Acq. Method Set: TGU n B
Date Processed: 3/1/2023 1:11:03 PM
Processing Method: SAWNUV353
Channel Name: 2487Channel 1
ColumnType 1HR,2HR,4HR

Sample Name	Retention Time (min)	MP (Daltons)	Mn (Daltons)	Mv (Daltons)	Mw (Daltons)	Mz (Daltons)	InVis	Mw/Mn	Area (µV*sec)	% Area	K (dl/g)	alpha
KTFT-100 isx.	14.989	43813881	20813122	54899171	64141909	146686996	17.015	3.08	53780	1.16	0.000111	0.670
KTFT-100 isx.	19.695	81326	29377	60650	69674	151103	0.178	2.37	3333420	71.99	0.000111	0.670
KTFT-100 isx.	24.776	3013	2196	3157	3305	4156	0.025	1.50	1242921	26.84	0.000111	0.670

Report Method: Overlay GPC pdf   Printed 1:22:53 PM 3/1/2023   Page: 1

Figure S5. The chromatogram of copolymer **IV** before heating (starting copolymer).

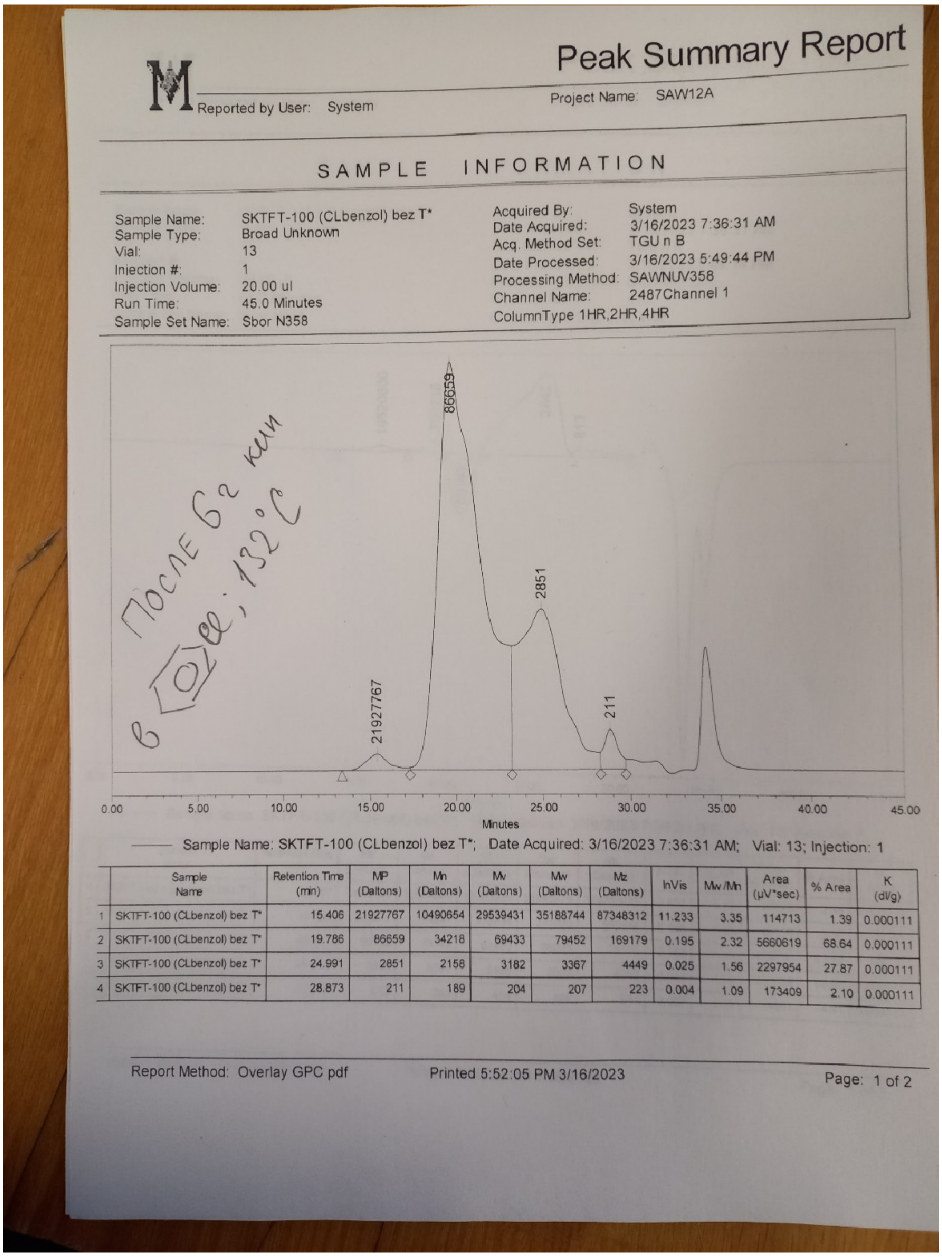
Peak Summary Report

Reported by User: System

Project Name: SAW12A

SAMPLE INFORMATION

Sample Name:	SKTFT-100 (CLbenzol) bez T*	Acquired By:	System
Sample Type:	Broad Unknown	Date Acquired:	3/16/2023 7:36:31 AM
Vial:	13	Acq. Method Set:	TGU n B
Injection #:	1	Date Processed:	3/16/2023 5:49:44 PM
Injection Volume:	20.00 ul	Processing Method:	SAWNUV358
Run Time:	45.0 Minutes	Channel Name:	2487Channel 1
Sample Set Name:	Sbor N358	ColumnType	1HR,2HR,4HR

Sample Name: SKTFT-100 (CLbenzol) bez T*; Date Acquired: 3/16/2023 7:36:31 AM; Vial: 13; Injection: 1

	Sample Name	Retention Time (min)	MP (Daltons)	Mn (Daltons)	Mv (Daltons)	Mw (Daltons)	Mz (Daltons)	InVis	Mw/Mn	Area (µV*sec)	% Area	K (dl/g)
1	SKTFT-100 (CLbenzol) bez T*	15.406	21927767	10490654	29539431	35188744	87348312	11.233	3.35	114713	1.39	0.000111
2	SKTFT-100 (CLbenzol) bez T*	19.786	86659	34218	69433	79452	169179	0.195	2.32	5660619	68.64	0.000111
3	SKTFT-100 (CLbenzol) bez T*	24.991	2851	2158	3182	3367	4449	0.025	1.56	2297954	27.87	0.000111
4	SKTFT-100 (CLbenzol) bez T*	28.873	211	189	204	207	223	0.004	1.09	173409	2.10	0.000111

Report Method: Overlay GPC pdf Printed 5:52:05 PM 3/16/2023 Page: 1 of 2

Figure S6. The chromatogram of copolymer IV after heating at 132°C in chlorobenzene at 6 hours.
